# Elimination of enzymes catalysis compartmentalization enhancing taxadiene production in *Saccharomyces cerevisiae*

**DOI:** 10.3389/fbioe.2023.1141272

**Published:** 2023-02-20

**Authors:** Chenglong Zhang, Wang Chen, Tianyu Dong, Ying Wang, Mingdong Yao, Wenhai Xiao, Bingzhi Li

**Affiliations:** ^1^ Frontier Science Center for Synthetic Biology, Key Laboratory of Systems Bioengineering (Ministry of Education), School of Chemical Engineering and Technology, Tianjin University, Tianjin, China; ^2^ Georgia Tech Shenzhen Institute, Tianjin University, Shenzhen, China

**Keywords:** taxadiene, catalysis compartmentalization, enzyme fusion, relocalization, *Saccharomyces cerevisiae*

## Abstract

Taxadiene is an important precursor in taxol biosynthesis pathway, but its biosynthesis in eukaryotic cell factories is limited, which seriously hinders the biosynthesis of taxol. In this study, it is found that there was the catalysis compartmentalization between two key exogenous enzymes of geranylgeranyl pyrophosphate synthase and taxadiene synthase (TS) for taxadiene synthesis progress, due to their different subcellular localization. Firstly, the enzyme-catalysis compartmentalization was overcome by means of the intracellular relocation strategies of taxadiene synthase, including N-terminal truncation of taxadiene synthase and enzyme fusion of GGPPS-TS. With the help of two strategies for enzyme relocation, the taxadiene yield was increased by 21% and 54% respectively, among them the GGPPS-TS fusion enzyme is more effective. Further, the expression of GGPPS-TS fusion enzyme was improved *via* the multi-copy plasmid, resulting that the taxadiene titer was increased by 38% to 21.8 mg/L at shake-flask level. Finally, the maximum taxadiene titer of 184.2 mg/L was achieved by optimization of the fed-batch fermentation conditions in 3 L bioreactor, which is the highest reported titer of taxadiene biosynthesis accomplished in eukaryotic microbes. This study provides a successful example for improving biosynthesis of complex natural products by solving the critical problem of multistep enzymes catalysis compartmentalization.

## 1 Introduction

Taxol is one of the best natural anti-tumor drugs on the market. It is considered to be one of the most effective anti-cancer drugs for human beings in the next 20 years (Yang et al., 2020; Huang et al., 2021). The main source of taxol is derived from taxus plants, which is limited by the taxol abundance and the natural resources of taxus plants. On natural conditions, the growth speed of taxus plants is slow, and regeneration ability is poor, thus the extracted taxol has been unable to meet the demand of clinical from taxus plants (Goldspiel, 1997; Htay and Liu, 2005; Miller et al., 2008). At present, the production of taxol mainly as and semi-synthetic raw material using the natural precursor 10-deacetylbaccatin III. However, the acquisition of 10-deacetylbaccatin III has some limitations, such as low extraction efficiency, high production cost and dependence of raw materials on taxus plants (Cragg, 1998; Baloglu and Kingston, 1999; Frense, 2007). With the help of microbial metabolic engineering and synthetic biology technology, constructing a suitable heterologous biosynthesis system to produce taxol gradually became a research hotspot (Choi et al., 2019; Zhang et al., 2022). In recent years, the biosynthesis of taxadiene, an important intermediate of taxol, has made some progress (Gallego-Jara et al., 2020; Sabzehzari et al., 2020; Hu et al., 2021).

Taxadiene is a diterpenoid compound derived from cyclized geranyl pyrophosphate (GGPP) substrate of taxadiene synthase (TS) (Hefner et al., 1998; Williams et al., 2000; Walker and Croteau, 2001). GGPP is a common precursor produced from MEP or MVA pathway and generated by geranyl diphosphate synthase (GGPPS) (Vranová et al., 2013; Liao et al., 2016; Chatzivasileiou et al., 2019). To date, taxadiene biosynthesis has been realized and modified in different microbial systems (Moser and Pichler, 2019; Navale et al., 2021). In 2010, Ajikumar *et al.* gained the yield of taxadiene of 300 mg/L in shake flask fermentation through engineering multi-module metabolic pathway and optimizing the metabolic balance of taxadiene synthesis. And then the yield of taxadiene was further increased to 1,020 mg/L by modifying fed-batch fermentation (Ajikumar et al., 2010). To realize the biosynthesis of taxol, taxadiene also needs to undergo a series of complex reactions such as hydroxylation, acetylation and epoxidation (Jennewein and Croteau, 2001; Hu et al., 2021). But the complete intimal system and protein post-translational modification system were lack in the prokaryotic system, which severely limits biosynthesis of taxol using prokaryotic system (Bheri et al., 2020; Ramazi and Zahiri, 2021). Thus, in the past decade, there has been no major breakthrough in the heterosynthesis of taxol in *E. coli* (*E. coli*). And the eukaryotic system was regarded as a potential candidate for heterosynthesis of taxol, since the eukaryotic system was suitable for the expression of multiple heterologous P450 hydroxylases and their reductase, which are necessary for taxol synthesis (Hauser and Matthes, 2017; Jia et al., 2022). Therefore, in recent years, eukaryotic systems have been increasingly favored by researchers in taxadiene biosynthesis. Engels *et al.* screened the sources of GGPPS enzyme, engineered the isoprene pathway and optimized the TS gene, achieving the yield of taxadiene of 8.7 mg/L, which laid a foundation for the production of taxol in *Saccharomyces cerevisiae* (*S. cerevisiae*) (Engels et al., 2008). Behnaz *et al.* enhanced the expression of TS by introducing the solubilizing tags to improve taxadiene titre to 57 mg/L at 30°C. Meanwhile, Behnaz *et al.* also found the temperature-dependant phenomenon of TS, according to that, a maximum taxadiene titre of 129 mg/L was obtained by manipulating the fermentation temperature at 20°C (Nowrouzi et al., 2020). In addition, Li *et al.* introduced taxadiene synthase, and taxadiene-5α-hydroxylase in Nicotiana benthamiana through chloroplastic compartmentalized metabolic engineering to produce taxadiene (56.6 μg/g FW) and taxadiene-5α-ol (1.3 μg/g FW). This also was an alternative eukaryotic platform for taxol production (Li et al., 2019). Previous studies have shown that the accumulation of GGPP is abundant enough to supply downstream metabolic pathways for the production of taxadiene and its derivatives in both prokaryotic and eukaryotic systems (Ober, 2010; Zhou et al., 2012). However, the synthesis efficiency of taxadiene in the eukaryotic system still needs to be further improved compared to that in the prokaryotic system. The eukaryotic cells have abundant subcellular organelle structures, which may lead to different localization of the heterologous proteins for complex natural products (Luo et al., 2015; Du and Li, 2021). Thus, we speculate that key enzymes catalysis compartmentalization may be an important factor to limit the taxadiene biosynthesis in eukaryotic system.

Focusing on the problem of enzymes catalysis compartmentalization, many researchers have done a lot of work and made great achievements. Dawid *et al.* truncated the N-terminal mitochondrial location sequences of valine biosynthesis enzymes Ilv2, Ilv5 and Ilv3 to relocate the truncated enzymes into the cytoplasm, resulting in the increase of isobutanol production (Brat et al., 2012). Jiang *et al.* employed synthetic protein scaffolds to colocalize the sequential enzymes of Idi1, GES-Erg20 and IS to eliminate enzymes catalysis compartmentalization, and the citronellol titer was incerased to 8.30 g/L (Jiang et al., 2021). In addition, protein fusion is also an important means to realize target gene relocation and eliminate enzymes catalysis compartmentalization. Ma *et al.* introduced the peroxisome targeting sequence SKL to fusion proteins CrtW-CrtZ, causing fusion proteins CrtW-CrtZ to relocate to suitable organelle of peroxisome, and the astaxanthin titer was increased to 58.7 mg/L (Ma et al., 2021). In addition, Shi *et al.* fused protopanaxadiol synthase (PPDS) to lipid droplet membrane protein Pln1 for closing the spatial distance to substrate dammarenediol-II (DD) accumulated in lipid droplet, leading to the final product ginsenoside Compound K yield of 5 g/L (Shi et al., 2021).

**FIGURE 1:**
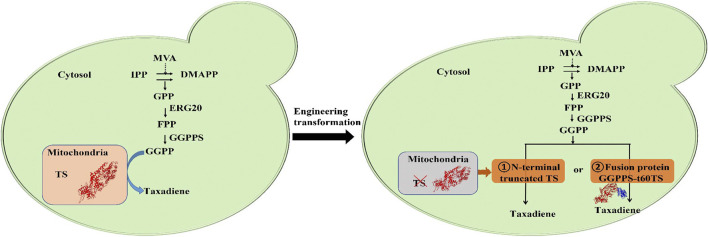
The biosynthesis pathway and metabolic engineering transformation of taxadiene. MVA, mevalonate; IPP, isopentenyl pyrophosphate; DMAPP, dimethylallyl pyrophosphate; GPP, geranyl diphosphate; FPP, farnesyl diphosphate; GGPPS, geranylgeranyl diphosphate synthase; GGPP, geranylgeranyl diphosphate; TS, taxadiene synthase.

In our previous study, we successfully constructed a high-yield strain of GGPP by screening the metabolic pathway gene and regulating the promoters of critical genes, which provided sufficient precursors for the biosynthesis of taxadiene (Song et al., 2017). Herein, we verified the catalysis compartmentalization of two key exogenous enzymes GGPPS and TS in taxadiene synthesis pathway (Figure 1). Next, we reconstructed the subcellular localization of TS in *S. cerevisiae* to eliminate enzymes (enzyme) catalysis compartmentalization using the methods of N-terminal signal peptide truncation of TS and enzyme fusion of GGPPS-t60TS, and the taxadiene yield was increased by 21% and 54% respectively. Especially, the GGPPS-TS fusion enzyme is more effective. And then, we improved the expression of GGPPS-TS fusion enzyme *via* the multi-copy plasmid, leading to the higher taxadiene titer of 21.8 mg/L at the shake-flask level. Finally, the taxadiene titer was increased to 184.2 mg/L for 144 h on the optimized fed-batch fermentation conditions in 3 L bioreactor, which is approximately increased to 17.8-fold compared with the initial strain yZCL010. This study is a successful example of improving the biosynthesis of complex natural products by overcoming multistep enzyme catalysis compartmentalization in the eukaryotic microbial cell.

## 2 Materiails and methods

### 2.1 Strains and culture media

All the yeast strains used in this study were listed in Table 1. *E. coli* Top10 (TransGen Biotech, Beijing, China) was used for plasmids construction and amplification. BY4742 used for construction of taxadiene producing strains (listed in Table 1), was obtained from EUROSCARF (Frankfurt, Germany) (Entian and Kotter, 2007).

**TABLE 1:** Yeast strains used in this study.

Strains	Description	Source
BY4742	MATα his3Δ1 leu2Δ0 lys2Δ0 ura3Δ0	Invitrogen
Sc01	Delta: *P*_TDH1_-*Sa*GGPPS	This lab
	-*P*_GPM1_-BTS1-ERG20-P_PDC1_-tHMGR; *LEU2*	
Sc02	Delta: *P*_TDH1_-*Sa*GGPPS	This lab
	-*P*_GPM1_-BTS1-ERG20-*P*_PDC1_-tHMGR; *LEU2; Ura3*	
yZCL004	Sc02; pZCL060; *Ura3*	This work
yZCL005	Sc02; pZCL055; *Ura3*	This work
yZCL006	Sc02; pZCL061; *Ura3*	This work
yZCL007	Sc02; pZCL062; *Ura3*	This work
yZCL010	Sc01; pZCL053	This work
yZCL011	Sc01; pZCL063	This work
yZCL012	Sc01; pZCL064	This work
yZCL047	Sc02; pZCL069; *Ura3*	This work
yZCL059	Sc01; pZCL081	This work
yZCL060	Sc01; pZCL082	This work
yZCL061	Sc01; pZCL083	This work

LB medium (0.5% yeast extract, 1% tryptone and 1% NaCl) was used to culture *E. coli* Top 10 for the propagation of recombinant plasmids, supplemented with 50 μg/mL kanamycin or 100 μg/mL ampicillin at 37°C. Yeast cells were routinely cultured in YPD medium (1% yeast extract, 2% peptone, 2% glucose) or synthetic complete (SC) drop-out medium (2% dextrose, 0.67% yeast nitrogen base, 0.2% amino acid mix lacking selected amino acids) at 30°C. The selected plasmids carrying nutritional screening tags were introduced into *S. cerevisiae* cells and cultured in a synthetic complete medium without corresponding amino acids. All the medium formulations for yeast culture was the same as our previous work (Zeng et al., 2020).

### 2.2 Plasmid construction

All the plasmids are summarized in Supplementary Table S1, and the primers used during plasmid construction are summarized in Supplementary Table S2. The yeast expression plasmid pRS415 and pRS425 were purchased from ADDGENE (American) and the ampicillin resistant gene of the plasmid was substituted by kanamycin resistant gene to construct pRS415K and pRS425K. *Sa*GGPPS gene (ACCESSION P39464) and *Tb*TS gene (ACCESSION Q41594) were codon optimized for expression in *S. cerevisiae* and synthesized by GenScript,Inc. (China) (Supplementary Table S3). The basic gene expression cassette was assembled by overlap extension PCR (OE-PCR) (pZCL026; pZCL027; pZCL041) (Supplementary Figure S2). The OE-PCR products were digested by *BamH* I/*Not* I and inserted into the corresponding sites of expression plasmid pRS415k, obtaining a series of plasmids for the construction of target enzymes localization strains. Then the plasmid with N-terminal truncated *Tb*TS or fusion protein GGPPS-TS (including pZCL063; pZCL064; pZCL071) were constructed based on pZCL041(Supplementary Table S1). The plasmids were transformed of into yeast strains by the LiAc/ssDNA carrier DNA/PEG3350 method. The *S. cerevisiae* strains constructed in this study were listed in Table 1(Gietz and Woods, 2006).

### 2.3 Fermentation conditions

To prepare seed vials, single isolates of each strain from agar plates were grown for SD Agar plate at 30°C and 200 rpm. And the preculture was transferred to a 250 mL flask filled with 50 mL fresh culture medium, the initial OD_600_ was 0.2. And then preculture was transferred into 250 mL flasks with 50 mL the same fresh medium with initial OD_600_ of 0.2 and cultivated at 30°C, 250 rpm for 10–12 h until the exponential phase. Finally, the third-grade seeds were transferred to a shake flask and fermented for 120 h. The cultured tertiary seeds were then transferred to 200 mL fresh SC medium for 10 h until OD_600_ reached about 6.0. Then the seed culture was transferred to a bioreactor containing a 2.7 L fermentation medium. The initial fermenter medium was YPD containing 20 g/L glucose. The 10% (v/v) n-dodecane (Sigma-Aldrich) was added to the culture at the beginning of the fermentation to enrich taxadiene, which could minimize the loss of taxadiene the product and protect the cells from phase the toxicity brought by taxadiene (Brennan et al., 2012).

For fed-batch fermentation, the inoculation amount in the fermenter is 10% (v/v), and the seed preparation conditions are the same as those in the shaking flask fermentation. The stirring speed is 300 rpm. The temperature was controlled at 30°C. Air flow is used to supply oxygen to the fermentation tank at 2 vvm, and the pH value in the fermentor was controlled at 6.0 with 3 M NaOH. In addition, the biomass in fermentation and the accumulation of taxadiene in a later stage were increased by nitrogen supplemental. In addition, 5 g/L nitrogen yeast extract was added three times at the initial stage of culture every 7 h during the beginning 48 h of cultivation. After 48 h of fermentation, the temperature dropped to 20°C, and 20% (v/v) dodecane was added for two-phase extractive fermentation, and Galactose inducer was added until the final concentration was 20 g/L. Cell growth and glucose concentration were continuously monitored during fermentation. The cell growth and the glucose concentration were constantly monitored during the fermentation process. 500 g/L glucose was fed periodically into the fermentation to keep the glucose concentration under 1.0 g/L. And the organic layer was harvested for taxadiene analysis by centrifugation of the fermentation broth at 12,000 rpm for 10 min. The GGOH (98% purity) was prepared to construct a standard curve to determine the GGOH yield.

### 2.4 Analytical methods

GC-TOF/MS analyzed taxadiene and GGOH in fermentation products. The target product extracted from n-dodecane was diluted with n-hexane, then 1 μL sample was injected into Shimazu GC-2030 using a Shimazu GCMS-QP2020 automatic sampler. The sample was detected using a quartz capillary column (30 m × 0.25 mm, 0.25 mm DB-5MS, J&W Scientific, Folsom). Design the relevant parameters of the sample detection method. The injector temperature was set at 260°C. The column effluents were introduced into the ion source (250°C) of TOF/MS. And ions were generated by 40 mA ionization current of a 70 eV electron beam. The mass scan range was 50–800 m/z.

For GC-TOF/MS analysis of taxadiene and GGOH, the column chamber temperature was first kept constant at 70°C for 1 min, then increased to 200°C at a rate of 30°C/min for 1 min. Next it increased to 265°C at a rate of 12°C/min and kept for 3 min. The total run time was 14.75 min. Taxadiene was identified by mass fragments 109 m/z and 122 m/z, and the peak time was 10.32 min. GGOH was identified by mass fragments of 69 m/z, 93 m/z, and 119 m/z, and the peak time was 11.35 min.

### 2.5 Assay of protein subcellular localization

Fluorescence microscopy was used to observe the distribution of fluorescent signals of target proteins in the subcellular to determine the subcellular localization of target proteins. A single colony of inverters extracted from an SC-U-L agar plate was first cultured into a 20 mL tube containing 5 mL SC-U-L medium and cultured at 30°C and 220 rpm for 20–24 h to achieve the exponential phase. The pre-cultured cells were then transferred to 20 mL test tubes and 5 mL identical fresh medium, initially OD_600_, and cultured at 30°C and 220 rpm for 48 h. Fluorescence image was observed under a fluorescence microscope and treated with fluorescence microscopy software FCSnap.

## 3 Results and Discussion

### 3.1 Subcellular localization of GGPPS and TS in taxadiene biosynthesis pathway

**FIGURE 2:**
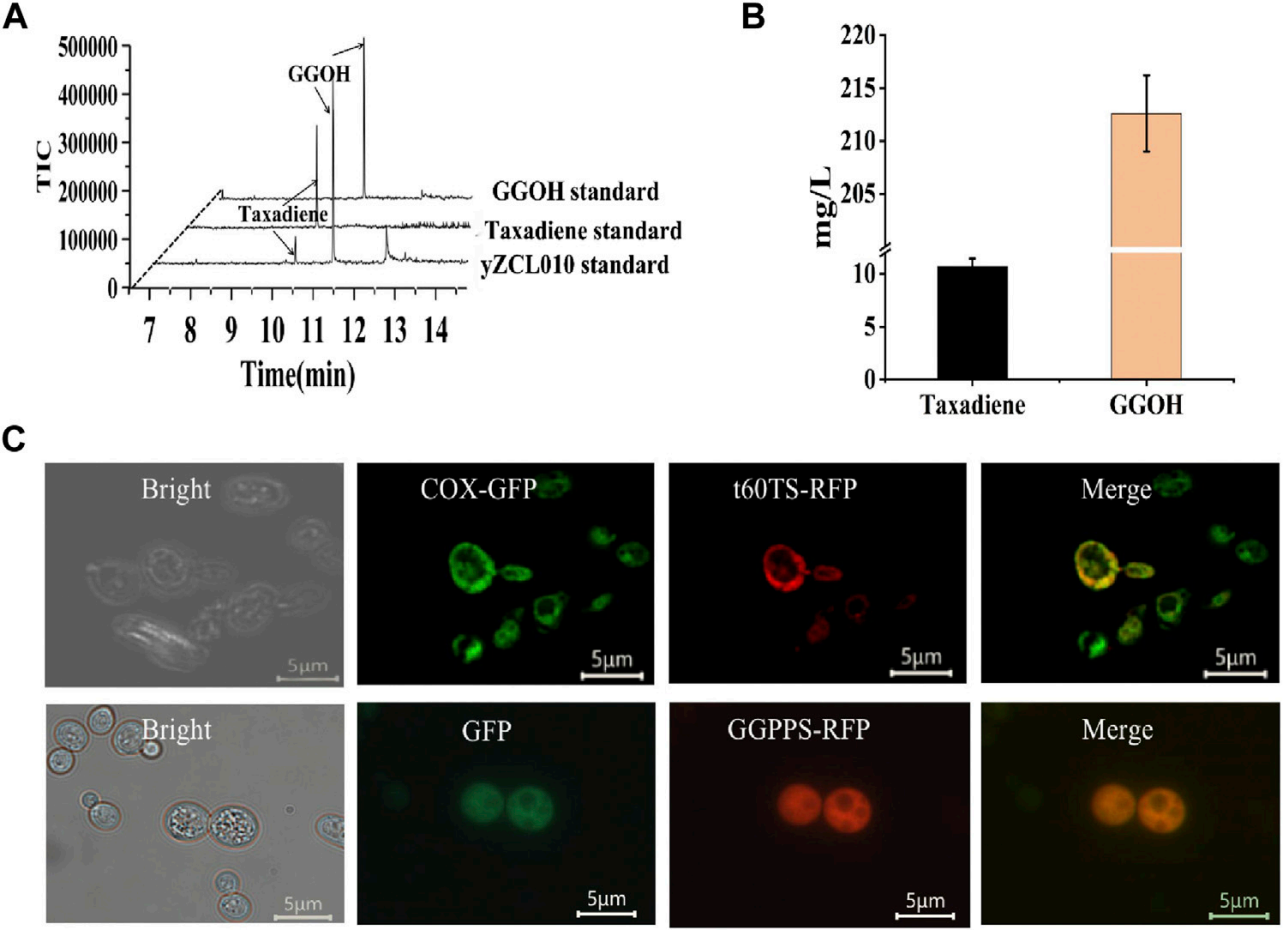
Construction of the initial taxadiene production strain and subcellular localization of key exogenous enzymes. **(A)** GC-MS results for the initial strain for taxadiene and GGOH production. The taxadiene and GGOH eluted at about 10.3 min and 11.3 min. **(B)** The titers of taxadiene and GGOH in strain yZCL010. **(C)** Subcellular localization of t60TS and GGPPS in *S. cerevisiae*.

In this study, Geranylgeranyl diphosphate synthase from *Sulfolobus acidocaldarius* (*Sa*GGPPS) and sequence-optimized TS from *Taxus brevifolia* (*Tb* t60TS) was introduced into the high-yield GGPP strain to construct the initial taxadiene production strain yZCL010 (Figure 2A). And the taxadiene titer of 10.2 mg/L and the geranylgeraniol (GGOH) titer of 214.2 mg/L were detected in the production strain yZCL010 (Figure 2B). According to the large accumulation of by-product GGOH in the synthesis of taxadiene, we speculated that the synthesis process of taxadiene with key enzymes of GGPPS and TS is limited, which should be the main reason for the low taxadiene production.

Whereafter, the subcellular localizations of two key enzymes *Tb*t60TS and *Sa*GGPPS for the taxadiene synthesis were detected. We firstly added red fluorescent protein (RFP) tag to the C-terminus of each target enzyme, and co-expressed them with fluorescent protein labeled endogenous subcellular localization protein, such as COXIII-GFP for mitochondrial localization characterization and sole expressed GFP for cytoplasmic localization characterization (Rodrigues et al., 2001; Naithani et al., 2003). Fluorescent microscopic image analysis of RFP and GFP signals showed that *Sa*GGPPS was located in cytoplasm, while t60TS was located in mitochondria (Figure 2C). These results indicate that GGPPS and t60TS localized at different subcellular, which would result in the problem of catalytic compartmentalization between the two sequent enzymes GGPPS and t60TS. Therefore the large amount of GGPP generated in the cytoplasm was difficult to contact with TS to produce taxadiene, due to GGPP hardly is transported through the plastid membrane with significant efficiency (Bick and Lange, 2003; Vranová et al., 2012). Instead, accumulated GGPP was converted into by-product GGOH. In our constructed the initial taxadiene production strain yZCL010, only less than 5% of the precursor GGPP was converted to the target product taxadiene, while almost all the rest was produced into by-product GGOH, which is a great metabolic drain. According to the above key enzymes localization analysis and product detection results, the catalytic compartmentalization of enzymes would reduce the availability of substrates and intermediates, leading to large accumulation of by-product and low yield of the target product.

### 3.2 Subcellular relocation of TS *via* N-terminal signal peptide truncation

From the above study, *Sa*GGPPS was located in the cytoplasm, which may lead to the accumulation of GGPP in the cytoplasm. Thus, *Tb*t60TS with mitochondria localization will hardly contact with the substrate GGPP to effectively generate taxadiene. Therefore, we attempted to relocate TS from mitochondria into the cytoplasm to improve conversion from GGPP to taxadiene. Here, the proper truncation position for *Tb*TS overexpressed in *S. cerevisiae* was attempted to relocate TS. Based on the predicted results on the secondary structure by https://bioinf.cs.ucl.ac.uk/psipred/ (Supplementary Figure S1A) (McGuffin et al., 2000), N-terminus of *Tb*TS was truncated at other two different positions (R84 and N97) besides M60 (t60TS), according to a certain gradient with slightly change, named t84TS and t97TS respectively (Supplementary Figure S1B).

**FIGURE 3:**
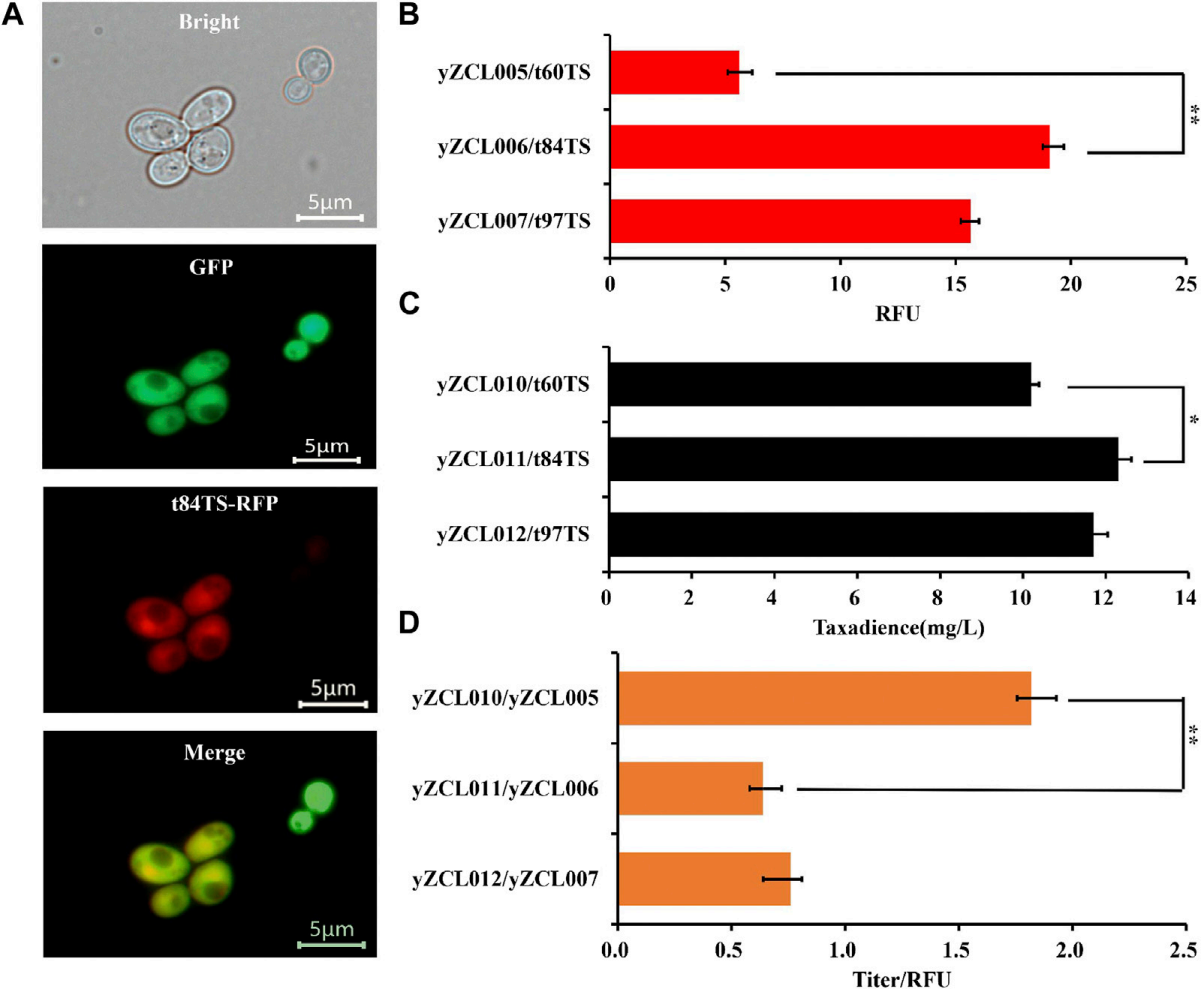
Effect of N-terminal truncation on subcellular localization and performance of TS in *S. cerevisiae*. **(A)** Subcellular localization of t84TS in *S. cerevisiae*. **(B)** Effect of N-terminal truncation on the expression of TS. **(C)** Effect of N-terminal truncation of TS on the yield of taxadiene. **(D)** Effect of N-terminal truncation on unit enzyme activity of TS. The error bars represent the means ± SD from three biological replicates. **p < 0.01, *p < 0.05, student’s *t*-test.

To determine the subcellular localization of the t84TS and t97TS in *S. cerevisiae*, RFP tag was applied to N-terminal truncated *Tb*TS and cytoplasmic localization protein GFP were also co-expressed (Refer to Section 3.1). Fluorescent microscopic image analysis showed that t84TS was located in the cytoplasm successfully (Figure 3A). In addition, t97TS were also located in the cytoplasm (data not shown).

Meanwhile, N-terminal truncated *Tb*TS (t84TS and t97TS) was introduced into the high-yield GGPP strain to construct the taxadiene production strain of yZCL011, and yZCL012, respectively. As shown in Figure 3B, notably t84TS and t97TS both led to an over 2.5-fold increase in protein soluble expression compared with that of t60TS, in view of the different fluorescence intensity detection. However, the titers of taxadiene only were slightly increased by 20.5% to 12.3 mg/L and by 14.3% to 11.7 mg/L in the production strains of yZCL011 and yZCL012 (Figure 3C), which indicated the taxadiene yield was not correspondingly increased with the enhanced expression of TS. In the case, we further investigated the effect of TS truncation on unit enzyme activity. It is finding that the enzyme activities of t84TS and t97TS with N-terminal deep truncation were either significantly decreased, even less than 50% (Figure 3D). Thus, the deep N-terminal truncation is an undesired strategy for optimizing the function of TS enzyme, even though *Tb*TS was relocate and increased on soluble expression.

### 3.3 Eliminate enzyme catalysis compartmentalization *via* protein fusion of GGPPS and TS

**FIGURE 4:**
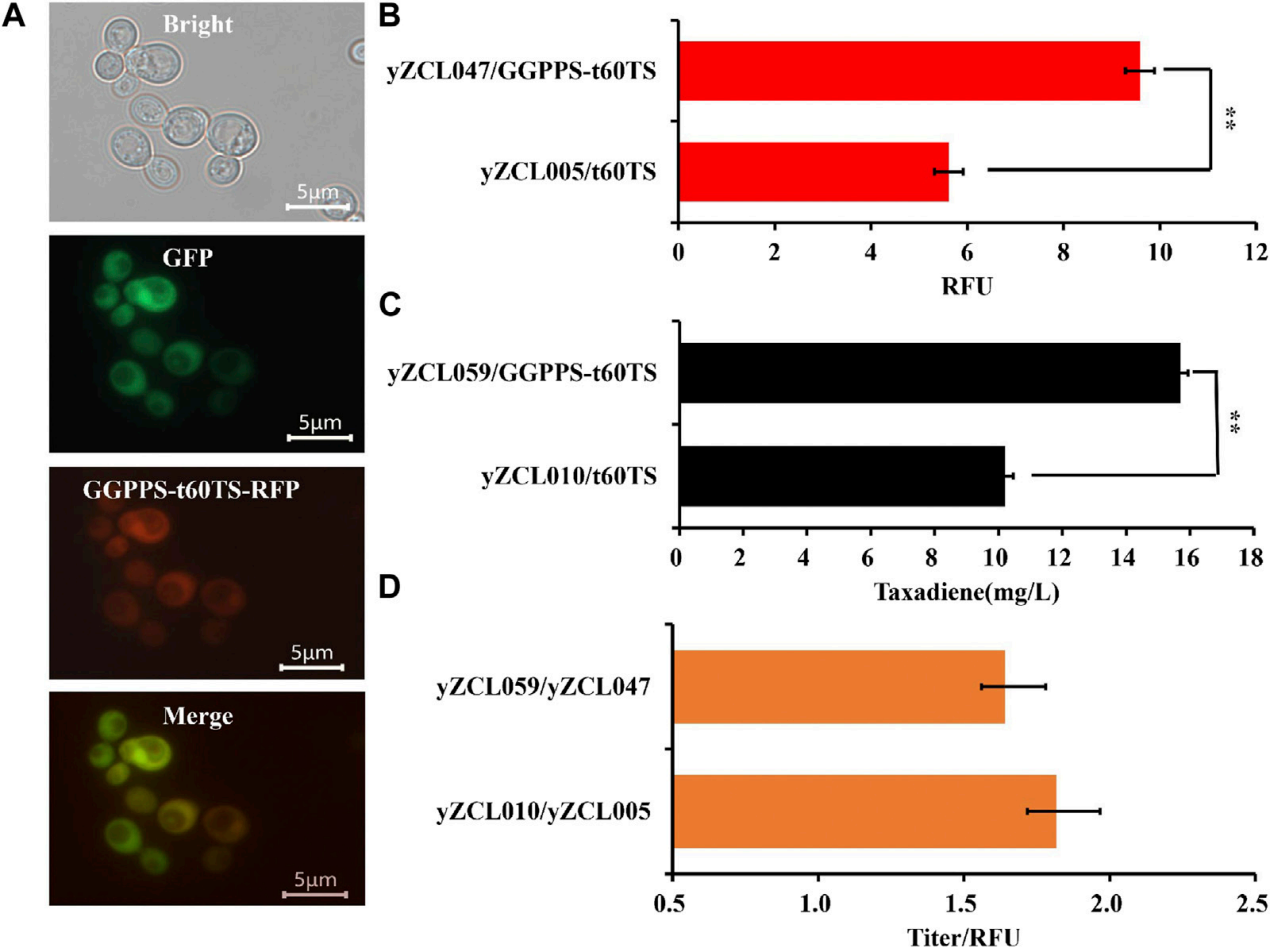
Effect of fusion protein GGPPS-t60TS on subcellular localization and performance of TS in *S. cerevisiae*. **(A)** Subcellular localization of fusion protein GGPPS-t60TS in *S. cerevisiae*. **(B)** Effect of fusion protein GGPPS-t60TS on the expression of TS. **(C)** Effect of fusion protein GGPPS-t60TS on the yield of taxadiene. **(D)** Effect of fusion protein GGPPS-t60TS on unit enzyme activity of TS. The error bars represent the means ± SD from three biological replicates. **p < 0.01, *p < 0.05, student’s *t*-test.

Since the catalysis compartmentalization between GGPPS and TS is the limiting step for taxadiene synthesis in *S. cerevisiae*, fusion expression of GGPPS and TS would overcome the problem of catalysis compartmentalization to improve sequential catalysis efficiency of GGPPS and TS. Here, the forward and reverse fusion of GGPPS and TS were adopted, and these two proteins were fused with the short flexible linker GSG (Ajikumar et al., 2010). Among them, in the forward fusion, *Sa*GGPPS pulled *Tb*t60TS enzyme to localize in the cytoplasm of *S. cerevisiae* (Figure 4A). Conversely, *Tb*t60TS would traction *Sa*GGPPS to localize in the mitochondria of *S. cerevisiae* in the reverse fusion.

Next, the forward and reverse fusion protein of GGPPS-t60TS and t60TS-GGPPS were introduced into the high-yield GGPP strain to construct the taxadiene production strain yZCL059 and yZCL060, respectively. In the two taxadiene production strains, the yields of taxadiene have been signally improved, reaching to 15.7 mg/L and 13.8 mg/L respectively, relying on the introduction of forward and reverse fusion proteins of GGPPS-t60TS and t60TS-GGPPS (Supplementary Figure S3). Moreover, applying the forward fusion protein of GGPPS-t60TS achieved more significant effect in enhancing taxadiene biosynthesis, and the taxadiene titer of strain yZCL059 was increased by 54%, compared with that of the initial strain yZCL010. To sum up, the forward and reverse fusion protein of GGPPS-t60TS and t60TS-GGPPS were located in cytoplasm and mitochondria of *S. cerevisiae* respectively, and the fusion protein of GGPPS-t60TS in cytoplasm had a greater advantage. It suggested that the cytoplasmic microenvironment seemed be more conducive to the synthesis pathway of taxadiene than mitochondria microenvironment, due to the membrane barrier effect of mitochondria restricting the transfer of intermediates. Moreover, the upstream MVA and FPP synthesis pathways are mainly distributed in the cytoplasm (Vranová E et al., 2013), which will Provide sufficient precursors for the synthesis of GGPP and taxadiene. This may also partly explain the reason of the high production of taxadiene in prokaryotes at present.

In addition, the soluble expression of GGPPS-t60TS was enhanced by about 60% than that of sole t60TS (Figure 4B), which indicates that GGPPS also contributed to the soluble expression of TS, similar to the soluble protein tag effect, in the forward fusion protein. This is a desirable outcome, since the heterologous expression of TS has not been satisfactory in the present study. As well, enzymes fusion in sequential catalysis progress (GGPPS and TS in this study) might minimize the distance between them for higher catalytic activity through enhancing GGPP accessibility for TS (Albertsen et al., 2011). According to the above two favorable effects, the GGPPS-t60TS raised the maximum yield of taxadiene of 15.7 mg/L in strain yZCL059 **(** Figure 4C). Meanwhile, there was no significant difference in unit enzyme activity between the fusion protein of GGPPS-t60TS and the initial t60TS (Figure 4D), which takes into account the intrinsic low enzyme activity of TS (Soliman and Tang, 2015). Therefore, the forward fusion protein of GGPPS-t60TS demonstrated the significant advantages in the efficient synthesis of taxadiene in the eukaryotic system.

### 3.4 Effect of increasing gene copy number on the yield of taxadiene

**FIGURE 5:**
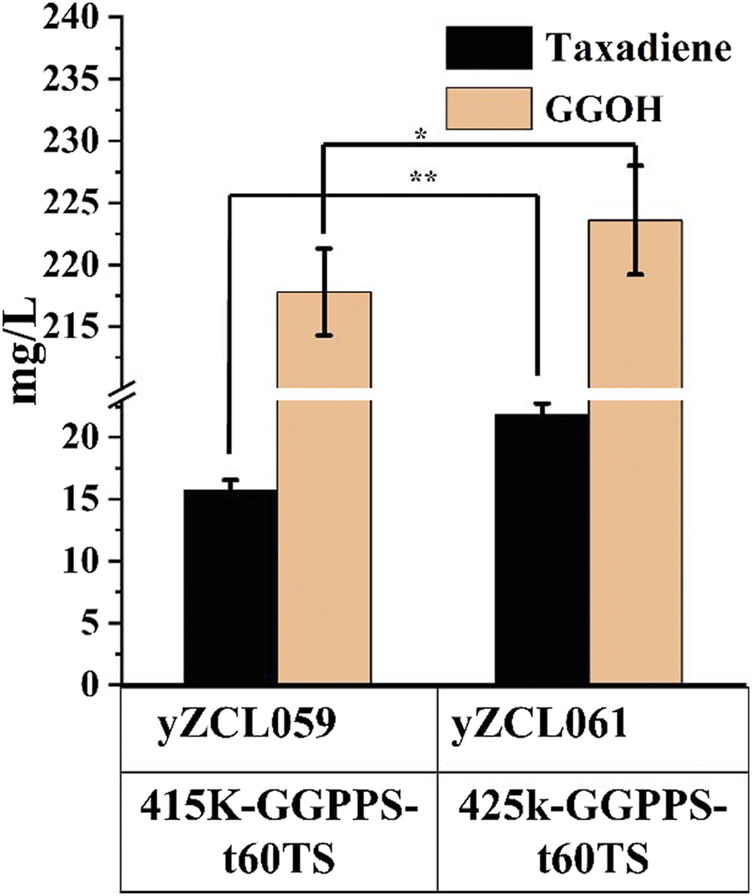
The effect of overexpression of fusion protein GGPPS-t60TS on the titers of taxadiene and GGOH. 415K incited single-copy plasmid, 425K incited multi-copy plasmid. The error bars represent the means ± SD from three biological replicates. **p < 0.01, *p < 0.05, student’s *t*-test.

The forward fusion protein GGPPS-t60TS was proved to be more benefit for taxadiene production. According to the poor soluble expression of TS in yeast on previous studies (Nowrouzi et al., 2020), the expression of the fusion protein GGPPS-t60TS module was enhanced using a multi-copy plasmid to improve the conversion rate of substrate GGPP to taxadiene. We firstly inserted the gene of fusion protein GGPPS-t60TS into the multi-copy plasmid PRS425K at restriction enzymes sites of *BamH* I and *Not* I, and then, the constructed multi-copy plasmid PRS425K -GGPPS-t60TS was introduced into the high-yield GGPP strain to obtained the taxadiene production strain yZCL061. As shown in Figure 5, the taxadiene yield of stain yZCL061was increased by 38.9% than that of stain yZCL059, benefiting from protein expression improvement by multiple-copy plasmid, and taxadiene titer reached to 21.8 mg/L in the shake flask. On the contrary, the by-product GGOH yield was also increased by 4% in the stain yZCL061 compared with that in the stain yZCL059, which did not show statistical significance. Ultimately, the titer ratio of taxadiene to GGOH is enhanced by over 108%, from the titer ratio of 4.7% in initial production strain yZCL010 to 9.8% in stain yZCL061 (Figure 2B, Figure 5). Therefore, the fusion protein of GGPPS-t60TS significantly promoted the conversion efficiency of target product taxadiene.

### 3.5 Taxadiene overproduction in fed-batch fermentation

**FIGURE 6:**
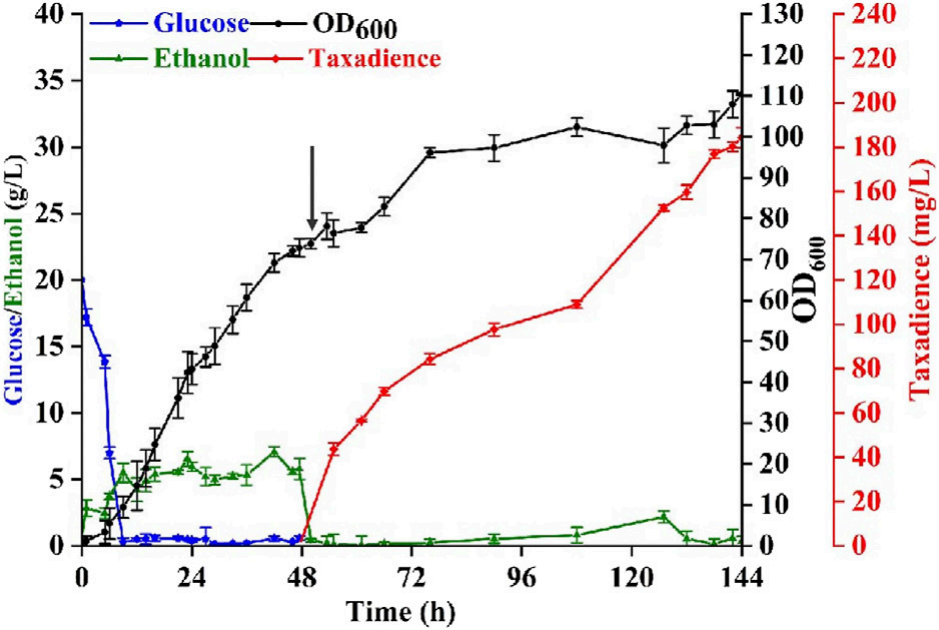
Taxadiene production in fed-batch fermentation. Fed-batch fermentations were performed at a 3.0 L scale, using YPD medium and by the engineered *S. cerevisiae* strain yZCL061. Black arrows represented the addition of n-dodecane and galactose into the medium. The error bars represent the means ± SD from three biological replicates.

To evaluate the performance of the best taxadiene biosynthesis strain (yZCL061), fed-batch fermentations were performed in a 5 L bioreactor using YPD as the batch medium. As shown in Figure 6, a total titer of 184.2 mg/L taxadiene with the maximal biomass at OD_600_ = 110.3 was achieved, which is the highest reported titer in eukaryotic cells. The fermentation process was divided into two stages. The first stage was the cell growth stage, in which the cultivation temperature was controlled at 30°C for 48 h until the cell density OD_600_ reached 72.8 (Figure 6). The initial glucose concentration was set as 20 g/L. The supplemental carbon source glucose was strictly controlled below 1 g/L due to the carbon source restriction strategy. The second stage taxadiene producing stage was initialized after the temperature was decreased to 20°C and 20 g/L galactose was added at 48 h (Nowrouzi et al., 2020). The supplemental carbon source was changed into ethanol because the existence of glucose would inhibit the expression of the GAL promoters. With fermentation proceeding, the taxadiene production also increased steadily. After 144 h cultivation, the taxadiene titer reached 184.2 ± 0.56 mg/L (Figure 6), which was eight times more than the output at the shake flask level. Even though the titer is still lower than that in *E. coli* (Ajikumar et al., 2010), increasing the yeast cell tolerance to taxadiene would be an efficient solution to minimize the taxadiene production gap between *E. coli* and yeast.

## 4 Conclusion

In this work, it is found that the catalysis compartmentalization between two key enzymes of GGPPS and TS limited taxadiene production. Applying the intracellular relocation strategies for TS eliminated catalysis compartmentalization *via* N-terminal truncation of TS and enzyme fusion of GGPPS-TS. With the help of forward fusion protein of GGPPS-t60TS, the taxadiene yield was increased by 54% to 15.7 mg/L. Subsequently, the taxadiene titer was further enhanced to 21.8 mg/L at shake-flask level by over-expressing fusion enzyme of GGPPS-t60TS in multi-copy plasmid. Eventually, a highest reported titer of 184.2 mg/L taxadiene in eukaryotic cells was achieved in 3 L fed-batch fermentation. Our study provides an effective strategy to eliminate multistep enzymes catalysis compartmentalization for improving biosynthesis of complex natural products.

## Data Availability

The original contributions presented in the study are included in the article/Supplementary Material, further inquiries can be directed to the corresponding author.
