# Supplementary material for: Elimination of enzymes catalysis compartmentalization enhancing taxadiene production in Saccharomyces cerevisiae
Source: Front Bioeng Biotechnol. 2023 Feb 20;11:1141272. doi: 10.3389/fbioe.2023.1141272 (PMC9986319; doi:10.3389/fbioe.2023.1141272)
Supplement: Supplementary file 1 [file DataSheet1.docx]

Supplementary Material

**Elimination of enzymes catalysis compartmentalization enhancing taxadiene production in *Saccharomyces cerevisiae***

**Chenglong Zhang^1^, Wang Chen^1^, Tianyu Dong^1^, Ying Wang^1^, Mingdong Yao^1*^,Wenhai Xiao^1,2^, Bingzhi Li^1^**

^1^ Frontier Science Center for Synthetic Biology and Key Laboratory of Systems Bioengineering (Ministry of Education), School of Chemical Engineering and Technology, Tianjin University, Tianjin 300072, China

^2^ Georgia Tech Shenzhen Institute, Tianjin University, Shenzhen 518071, China

*Correspondence:

Mingdong Yao

Email: [mingdong.yao@tju.edu.cn](mailto:mingdong.yao@tju.edu.cn)

**Table S1. Plasmids used in this study.**

| **Plasmid** | **Description** | **Source** |
| --- | --- | --- |
| pRS416 | Amp; URA3 | This lab |
| pRS415K | substitution of kanamycin resistant gene for ampicillin resistant gene based on pRS415 | This lab |
| pRS425K | substitution of kanamycin resistant gene for ampicillin resistant gene based on pRS425 | This lab |
| pZCL026 | *pRS415K-T_tdh1_*-GAL1/10-BsaI*-*RFP-*T_fba1_* | This study |
| pZCL027 | *pRS416-T_tdh1_*-GAL1/10-BsaI*-*GFP-*T_fba1_* | This study |
| pZCL041 | *pRS415K-T_tdh1_*-GAL1/10-BsaI-*T_fba1_* | This study |
| pZCL042 | *pRS425K-T_tdh1_*-GAL1/10-BsaI-*T_fba1_* | This study |
| pZCL051 | *pRS416-T_tdh1_*-GAL1/10*-*COXIII*-*RFP-*T_fba1_* | This study |
| pZCL052 | *pRS416-T_tdh1_*-GAL1/10-GFP-*T_fba1_* | This study |
| pZCL053 | *pRS415K-T_tdh1_*-GAL1/10-Tbt60TS-*T_fba1_* | This study |
| pZCL055 | *pRS415K-T_tdh1_*-GAL1/10-Tbt60TS*-*RFP-*T_fba1_* | This study |
| pZCL060 | *pRS415K-T_tdh1_*-GAL1/10-SaGGPPS*-*RFP-*T_fba1_* | This study |
| pZCL061 | *pRS415K-T_tdh1_*-GAL1/10-Tbt84TS*-*RFP-*T_fba1_* | This study |
| pZCL062 | *pRS415K-T_tdh1_*-GAL1/10-Tbt97TS*-*RFP-*T_fba1_* | This study |
| pZCL063 | *pRS415K-T_tdh1_*-GAL1/10-Tbt84TS-*T_fba1_* | This study |
| pZCL064 | *pRS415K-T_tdh1_*-GAL1/10-Tbt97TS-*T_fba1_* | This study |
| pZCL069 | *pRS415K-T_tdh1_*-GAL1/10*-*SaGGPPS-Tbt60TS-RFP-*T_fba1_* | This study |
| pZCL070 | *pRS415K-T_tdh1_*-GAL1/10*-*Tbt60TS-SaGGPPS-RFP-*T_fba1_* | This study |
| pZCL081 | *pRS415K-T_tdh1_*-GAL1/10-SaGGPPS-Tbt60TS*-T_fba1_* | This study |
| pZCL082 | *pRS415K-T_tdh1_*-GAL1/10-Tbt60TS-SaGGPPS*-T_fba1_* | This study |
| pZCL083 | *pRS425K-T_tdh1_-GAL1/10-*Sa*GGPPS-*Tb*t60TS-T_fba1_* | *This study* |

**Table S2 Primer used in this study.**

| **Primer name** | **Sequence (5′-3′)** |
| --- | --- |
| RFP-BsaI-F | CCGGTCTCCTGAAGGGTCCGGAGTTTCAAAAGGTGAAGAAGATAATATGGCTATTAT |
| RFP-BsaI-R | CTTCACCTTTTGAAACTCCGGACCCTTCAGGAGACCGGTCTCCCATTTATTATTG |
| P_tdh1_-F | GATCCACTAGTTCTAGAGCGGCCGCTTTACTCTATAGTACCTCC |
| T_fba1_-R | CTCCACCGCGGTGGCGGCCGCATAACTGACTCATTAGACACTTTTTGAAGC |
| Tbt60TS-RFP-F | CTAATACTATAACATACAATAATAAATGTCATCATCAACCGGTACTTCAAAGG |
| Tbt60TS-RFP-R | CCTTTTGAAACGGGTCCGGAAACTTGGATAGGGTCAATATAGACC |
| t84TS-RFP-F | ATAACATACAATAATAATGTCTGCTAACTACCACGGAGATTTG |
| t84TS-RFP-R | CCTTTTGAAACGGGTCCGGAAACTTGGATAGGGTCAATATAGACC |
| t97TS-RFP-F | TACTATAACATACAATAATAATGATACAAACATTGGAGACACCT |
| t97TS-RFP-R | CCTTTTGAAACGGGTCCGGAAACTTGGATAGGGTCAATATAGACC |
| t109TS-RFP-F | TACTATAACATACAATAATAATGACATACCAGGAGAGGGCC |
| t109TS-RFP-R | CCTTTTGAAACGGGTCCGGAAACTTGGATAGGGTCAATATAGACC |
| GGPPS-Tbt60TS-RFP-F | CTATAACATACAATAATAAATGTCTTACTTCGACAACTACTTTAACG |
| GGPPS-Tbt60TS-RFP-R | CCTTTTGAAACGGGTCCGGAAACTTGGATAGGGTCAATATAGACC |
| Tbt60TS-GGPPS-RFP-F | CTAATACTATAACATACAATAATAAATGTCATCATCAACCGGTACTTCAAAGG |
| Tbt60TS-GGPPS-RFP-F | TCACCTTTTGAAACTCCGGACCCTTTCCTTCTTCTAATGGTAAACTCTGCCAAATAC |
| GGPPS-Tbt60TS-F | CGTCAAGGAGAAAAAACTATATGTCTTACTTCGACAACTACTTT |
| GGPPS-Tbt60TS-R | AGTAACTTAAGGAGTTAAATTTAAACTTGGATAGGGTCAATATAGACC |

**Table S3 The Codon-optimized sequences of genes involved in this study.**

| **Genes** | | **Sequences** | |
| --- | --- | --- | --- |
| *Tbt60TS from Taxus brevifolia* | atgtcatcatcaaccggtacttcaaaggtagtatcagagacttcttctaccatagtagatgatatcccaagattgtctgctaactaccacggagatttgtggcatcacaacgtaatacaaacattggagacaccttttagagagtcttctacataccaggagagggccgatgaattggtcgtcaagatcaaagatatgtttaacgcattaggtgacggtgatatctcaccttctgcctatgacacagcttgggttgctagattggccacaatttcttctgatggatctgaaaagcctaggtttcctcaggcattaaactgggtctttaacaatcaattgcaagacggttcatggggtatagagtctcacttttctttatgcgatagattattgaacactacaaactcagtaatcgctttatctgtctggaaaaccggacactcacaggttcaacagggtgccgagttcatagccgaaaatttgaggttattaaacgaggaagatgagttatctcctgacttccaaatcatattcccagctttgttacagaaagccaaggctttgggtattaatttgccttacgacttgccattcattaaatacttgtcaaccactagggaagctaggttaactgacgtatcagctgccgccgataatatccctgctaatatgttgaatgccttagagggattggaggaagtaatagactggaacaaaatcatgagatttcaatctaaggacggatcattcttgtcttcacctgcatcaactgcttgcgttttgatgaataccggagatgagaagtgctttacattcttaaacaacttattggataagttcggaggatgtgtcccttgtatgtattctatcgacttgttggagaggttgtctttagtcgacaacatagaacacttaggtattggtaggcactttaagcaggaaattaagggagctttggattatgtctacagacactggtcagaaagaggaatcggttggggtagagactcattagtaccagatttaaacaccacagcattgggattgagaacattaaggatgcatggatataacgtttcttcagatgttttgaataatttcaaagacgagaacggtagattcttttcatctgccggtcagacacacgtcgaattaagatcagtagtaaacttgtttagagcctcagatttggccttcccagacgaaagagccatggatgacgctaggaagttcgctgaaccttacttgagagaggcattggcaactaagatctctactaacacaaaattgttcaaggaaatcgaatacgtcgttgagtacccatggcatatgtcaataccaaggttggaggcaaggtcttacatagactcttatgacgataactatgtttggcagagaaagacattgtatagaatgccatctttgtctaactcaaagtgtttggaattagccaaattggattttaacatcgtacaatcattgcaccaggaagaattaaagttattgactagatggtggaaggaatcaggtatggccgacataaacttcacaagacatagggtagccgaggtttacttctcttcagctactttcgagccagagtattctgctacaaggattgccttcaccaaaataggatgtttacaggtcttgttcgacgatatggcagacatttttgccactttggacgaattgaagtcattcaccgagggtgttaagagatgggatacttctttgttgcacgaaataccagagtgtatgcaaacatgctttaaggtctggtttaagttaatggaagaggtcaacaacgatgtagtcaaagtccaaggaagagacatgttagctcacattagaaaaccatgggagttatactttaactgctacgttcaagaaagggagtggttagaggctggttacatccctacttttgaggaatatttgaaaacctacgctatttcagtcggtttaggaccttgtacattgcagcctatcttattgatgggagagttagtaaaggacgacgttgtagaaaaggtccactacccttctaatatgttcgagttggtctcattgtcatggagattgacaaacgacaccaagacttatcaggccgagaaggctagaggacaacaagcatctggaatcgcatgttacatgaaggataacccaggtgccacagaagaggacgctatcaagcatatttgcagagtagttgacagggccttaaaagaagcctcattcgagtatttcaagccttcaaatgacatacctatgggttgtaaatcatttatcttcaacttaagattatgcgttcagatattttacaaattcatagatggatacggtattgctaacgaagagataaaagactatataaggaaggtctatattgaccctatccaagtttaa | | |
| GGPPS from *Sulfolobus acidocaldarius* | | | atgtcttacttcgacaactactttaacgagatcgtcaattcagtcaacgacatcatcaagtcatacatttctggagacgttcctaagttgtacgaagcctcatatcatttgttcacttctggtggaaaaaggttgaggccattgatattgacaatctcatcagatttgtttggtggacagagagaaagggcttattatgctggtgccgctatagaagtcttgcatactttcactttggttcacgatgacattatggaccaggataatatcagaaggggtttgccaactgttcatgtcaaatatggtttacctttggctatcttagccggagacttgttgcatgctaaagcatttcaattgttaacccaggctttgagaggattgccatctgagaccatcatcaaggctttcgatattttcaccaggtcaattatcatcatttcagagggtcaggccgtcgacatggaatttgaggataggatagacataaaggagcaggaatacttggatatgatttcaagaaagactgctgccttattttctgcttcatcatctattggtgctttaattgcaggtgctaacgacaacgatgttaggttgatgtctgattttggaacaaacttgggaatcgcttttcagattgtagacgatatcttgggtttgactgctgatgaaaaggaattgggaaagccagtcttctctgacataagggagggaaagaagacaattttagtaatcaagaccttggaattgtgcaaggaagatgagaaaaagatcgtattaaaggccttgggtaataagtctgcatctaaagaggagttaatgtcttcagccgacatcatcaaaaagtactcattggactacgcctacaatttggccgaaaaatactacaagaatgccatagactctttgaaccaggtttcttcaaagtcagatatccctggaaaggcattgaagtatttggcagagtttaccattagaagaaggaaataa |


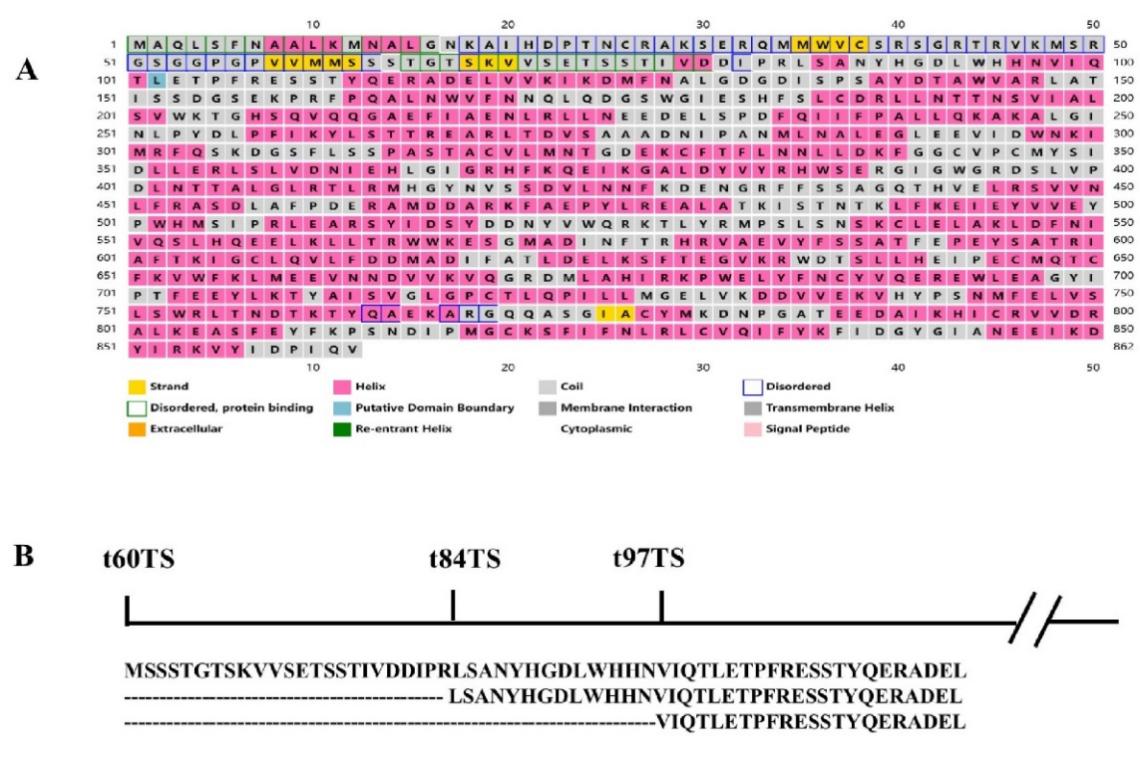


**Figure S1.** Secondary structural analysis and modification of *Tb*TS protein. **(A)**Feature predictions of *Tb*TS by PSIPRED protein structure prediction server (http://bioinf.cs.ucl.ac.uk/psipred/). **(B)** Schematic of the truncated position of N-terminus of *Tb*TS.


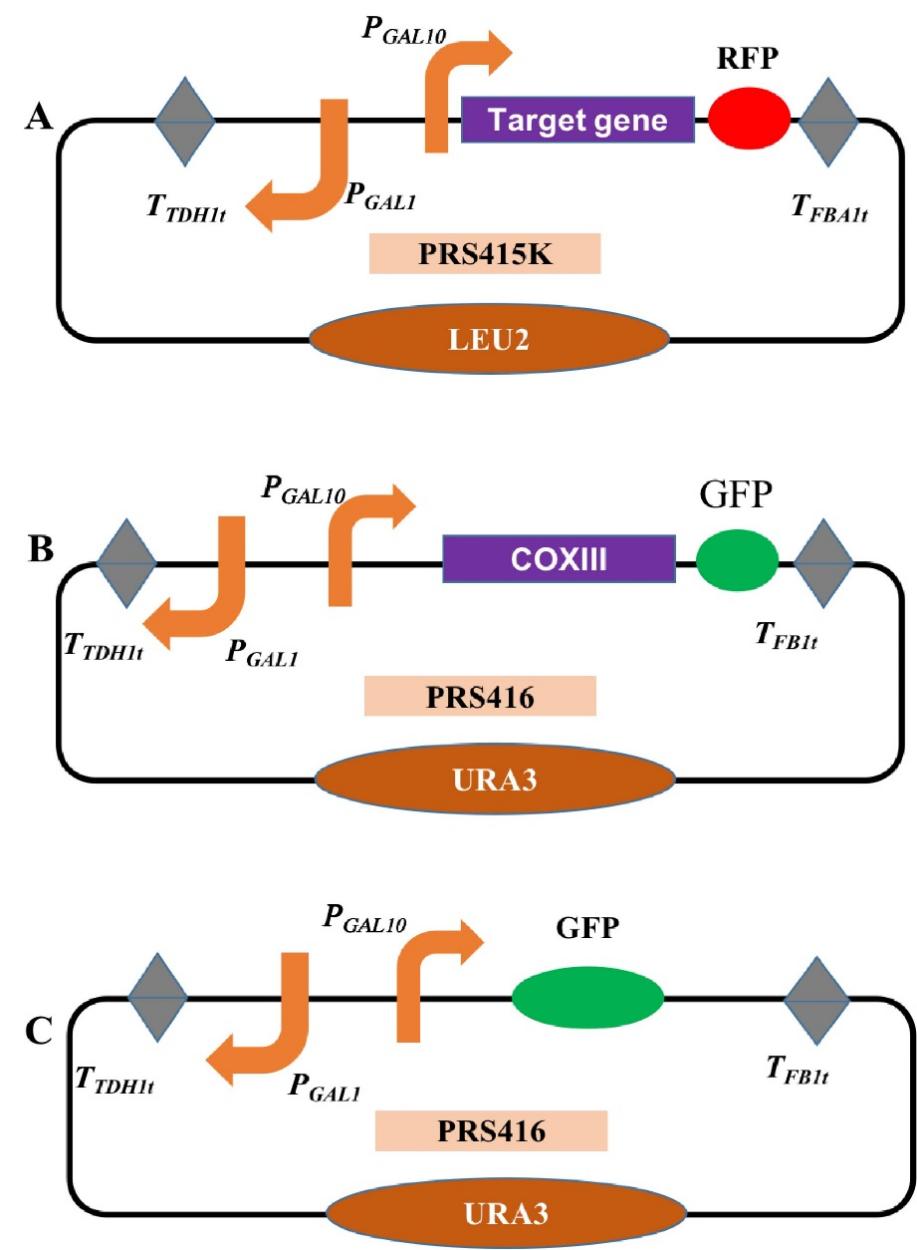


**Figure S2.** The construction of the localization modules (**A**) The expression plasmids for target gene localization module. (**B**) The expression plasmids for mitochondrial localization module. (**C**) The expression plasmids for cytoplasm localization module.


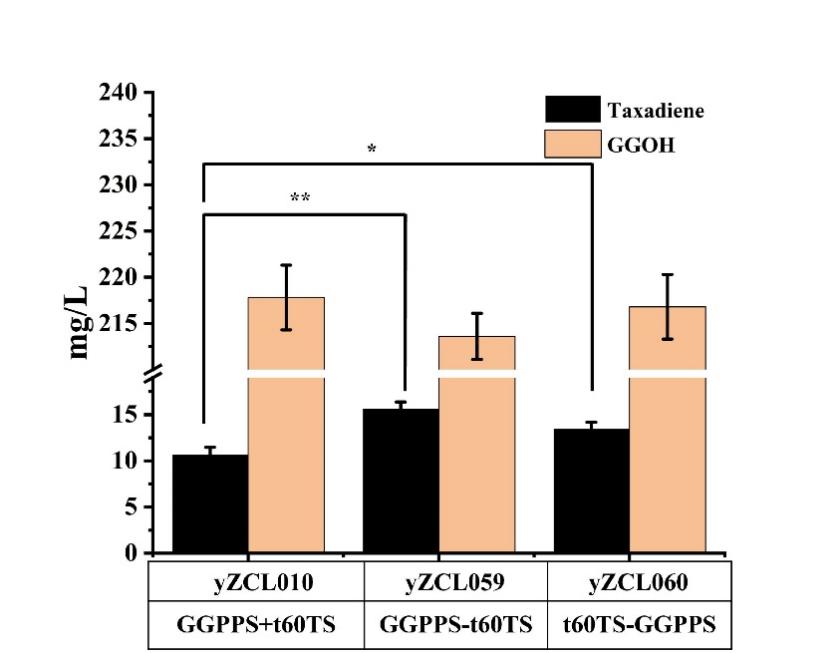


**Figure S3.** The effect of the forward and reverse fusion protein of GGPPS-t60TS and t60TS-GGPPS on taxadiene titer. All values presented are the mean of three biological replicates ± standard deviation. **P < 0.01, *P < 0.05, student’s t-test.
